# Outcome analysis of 215 patients with parotid gland tumors: a retrospective cohort analysis

**DOI:** 10.1186/s40463-015-0097-z

**Published:** 2015-10-29

**Authors:** Boban M. Erovic, Manish D. Shah, Guillem Bruch, u Johnston, John Kim, Brian O’Sullivan, Bayardo Perez-Ordonez, Ilan Weinreb, Eshetu G. Atenafu, John R. de Almeida, Patrick J. Gullane, Dale Brown, Ralph W. Gilbert, Jonathan C. Irish, David P. Goldstein

**Affiliations:** Department of Otolaryngology-Head and Neck Surgery, Wharton Head and Neck Program, University Health Network, Princess Margaret Cancer Centre, Toronto, ON Canada; Department of Radiation Oncology, Princess Margaret Cancer Centre, University of Toronto, Toronto, ON Canada; Department of Pathology, University Health Network, Princess Margaret Cancer Centre, Toronto, ON Canada; Department of Biostatistics, University Health Network, Princess Margaret Cancer Centre, Toronto, ON Canada; Princess Margaret Hospital, Wharton Head and Neck Centre, 610 University Avenue, 3rd Floor, Toronto, ON M5G 2 M9 Canada

**Keywords:** Prognostic factors, Salivary gland tumors, Periparotid lymph node metastases

## Abstract

**Background:**

To identify prognostic factors in patients with parotid gland carcinomas who were treated at the Princess Margaret Hospital.

**Methods:**

Clinical outcome of two hundred fifteen patients with malignancies of the parotid gland was evaluated over a 16-year period.

**Results:**

Two-hundred-fifteen patients with adenoid cystic carcinoma (*n* = 20), adenocarcinoma (*n* = 19), acinic cell carcinoma (*n* = 62), basal cell adenocarcinoma (*n* = 7), carcinoma-ex-pleomorphic adenoma (*n* = 18), mucoepidermoid carcinoma (*n* = 70) and salivary duct carcinoma (*n* = 19) have been included. The 5- and 10-year overall and disease-free survivals were 80.62 %/69.48 % and 74.37 %/62.42 %, respectively. Multivariable analysis showed that age greater than 60 years, advanced pN classification, histopathological grade and the presence of lymphovascular invasion significantly worsened overall and disease-free survival. Univariable analysis revealed periparotid lymph node involvement was associated with decreased overall (*p* < 0.0001) and disease-free survival (*p* < 0.0001).

**Conclusions:**

In addition to age, pN classification, histopathological grade, perineural invasion, and lymphovascular involvement, periparotid lymph node metastasis appears to be an important prognosticator in parotid gland malignancy.

## Introduction

Malignant salivary glands tumors are rare, representing only 2 % of all head and neck malignancies [1]. Salivary gland carcinomas represent a heterogeneous group of malignancies with diverse biological behaviors [2, 3], rendering standardization of management extremely difficult. In the first large published case series of 2807 patients with salivary gland malignancies over a 35 year period, Spiro [3] reported that the site of origin, histologic subtype, grading, and clinical stage were significant prognostic factors for overall survival. Wahlberg *et al.* [4] analyzed a Swedish cohort of 2465 patients treated between 1960 and 1998 for malignant parotid tumors and found that histopathological subtype, age and sex were also significant clinical predictors for survival [4]. Other studies have demonstrated the importance of regional lymph node involvement, positive surgical margins, perineural invasion, and facial nerve palsy as significant clinical predictors of outcome [4–7]. Recent studies have investigated molecular prognosticators associated with less favorable outcomes in those with salivary gland malignancy [8–11]. Interpretation of the literature is often difficult as patients in a given case series have typically been treated over extended periods of time, and using non-uniform treatment modalities [12].

The primary objective of this study was to analyze the outcome and patterns of failure in 215 patients with malignant parotid gland tumors managed at the Princess Margaret Cancer Centre (Toronto, Canada). The secondary objective was to evaluate whether previously reported clinical and pathologic factors were significant predictors of survival.

### Material and patients

A retrospective review of 215 consecutive patients with primary parotid gland cancers treated at the Princess Margaret Cancer Center between 1989 and 2005 was performed. Patients were identified through the Princess Margaret Cancer Registry and cross-referenced with a head and neck surgical registry. Approval was obtained from the Institutional Research Ethics Board prior to data collection. Patients with a newly diagnosed malignancy arising within the parotid were included in the study if they received some or all of their treatment at the Princess Margaret. A subset of patients included were those that had their initial surgery at an outside institution that were referred in shortly after their initial procedure for either further resection followed by radiation or for post-operative radiation alone. Patients with submandibular, sublingual and minor salivary gland cancers, lymphomas or malignancies metastatic to the salivary glands were excluded. Patients were also excluded if they were treated with palliative intent.

The management approach at the Princess Margaret for patients with parotid gland malignancy has been surgical resection with adjuvant radiotherapy used in those patients with positive margins, high-grade histology, perineural invasion/spread or nodal metastases, or where uncertainty existed about completeness of resection, usually arising from very close juxtaposition of the tumor to the facial nerve. Generally, in cases where the tumor was abutting but not invading the facial nerve and nerve function was normal pre-operatively, the facial nerve was preserved with the addition of post-operative radiotherapy. Therapeutic neck dissections were performed when there was clinical or radiographic evidence of nodal metastases. In patients without any evidence of nodal metastases, prophylactic neck dissection was performed in those patients with high-grade malignancies. For all patients undergoing surgery, the surrounding lymph nodes were examined, including the upper neck in parotid tumors. Enlarged nodes were sampled and if frozen section examination confirmed metastases, an appropriate neck dissection was performed. Patients that had an initial surgery at an outside center were offered revision surgery prior to post-operative radiotherapy if they had residual disease on MRI, otherwise they were managed with post-operative radiotherapy alone.

Diagnosis of all tumors was performed by head and neck pathologists and classified according to the World Health Organization (WHO) classification of salivary gland malignancies [2]. Demographic, clinical, and pathological data was obtained from hospital records. The pathological parameters included histologic subtype, perineural invasion (PNI), lymphovascular invasion (LVI), margin status, extra capsular extension and metastases to the peri-parotid lymph nodes. Peri-parotid and intra-parotid lymph nodes were defined as those nodes attached to or within the parotid gland, respectively. The grade of the tumour when reported by the pathologist was recorded. Disease was staged at the time of initial presentation using the American Joint Committee on Cancer (AJCC) classification staging system.

### Statistical analysis

Descriptive statistics were used for describing patient demographics and pathological characteristics. Categorical variables were expressed as counts and proportions, whereas continuous variables were expressed as means with standard deviations (SD). Outcome measures included control rates, overall survival (OS) and disease-free survival (DFS), which were estimated using the Kaplan-Meier product method. Time to event outcomes were calculated from the date of diagnosis to the event of interest. Differences between survival curves were analyzed using the log-rank test.

Potential prognostic variables achieving significance level of 0.20 or less on univariable analysis were subsequently entered into a multivariable Cox-proportional hazards model and stepwise model-building was used to determine the simplest model that best described the association in the data. Histologic subtype was not incorporated into the multivariable analysis for either OS or DFS as this would lead to excessive stratification of the data given the number of patients in the study. Grade was included in the multivariable analysis; however, since the majority of patients were either low or high grade, the intermediate grade patients were grouped with the high-grade patients. All *p*-values were 2-sided. Results were considered significant if *p* < 0.05. Statistical analyses were performed using SAS (Version 9.3, SAS Institute, Inc., Cary, NC).

## Results

A total of 215 patients with parotid gland cancers managed with primary surgery were included in the study. The mean (median) age of the patients was 55 (56) years (range 15–91) and 112 patients (52 %) were female. Out of the 215 patients only 12 (5.6 %) patients presented with a facial paralysis.

Facial nerve preserving surgery was performed in 179 patients with 28 patients undergoing a total parotidectomy with nerve sacrifice and an additional 8 had a total parotidectomy, nerve sacrifice and temporal bone resection. Adjuvant post-operative radiotherapy was given to 168 (78 %) patients. The mean and median radiation dose was 58 and 60 Gy, respectively (range from 35 to 70Gy). Neck dissections were performed in 105 (48.8 %) patients. Selective and modified radical neck dissection was performed in 81 (37.7 %) and 19 (8.8 %) patients, respectively. An additional 90 patients that did not have planned neck dissection had pathologic assessment of the intraparotid or periparotid lymph nodes.

Tumor characteristics, including T and N classification, are summarized in Table 1. Mucoepidermoid carcinoma (MEC) was the most common histologic variant of parotid gland cancer, accounting for 32.5 % percent of cases, followed in frequency by acinic cell (28.8 %) and adenoid cystic carcinoma (ACC) (9.3 %). Histopathological grade for each histologic subtype of parotid cancer, if specified is presented in Table 2.

**Table 1:** Demographic and clinicopathological data of 215 patients with major salivary gland carcinoma

Variable		Number of patients (%)
		Total = 215
Morphology	Acinic carcinoma	62 (28.84 %)
	Adenocarcinoma	19 (8.84 %)
	Adenoid cystic carcinoma	20 (9.30 %)
	Basal cell adenocarcinoma	7 (3.26 %)
	Carcinoma-ex pleomorphic	18 (8.37 %)
	Mucoepidermoid carcinoma	70 (32.56 %)
	Salivary duct carcinoma	19 (8.84 %)
Pathological Tumor classification	T1	68 (34.17 %)
	T2	61 (30.65 %)
	T3	48 (24.12 %)
	T4a	20 (10.05 %)
	T4b	2 (1.01 %)
	na	16 (7.44 %)
Pathological Lymph node status	N0	126 (70.79 %)
	N+	52 (29.21 %)
	na	37 (17.2 %)
Pathological Staging	I	64 (33.51 %)
	II	46 (24.08 %)
	III	40 (20.94 %)
	IVa	41 (21.47 %)
	na	24 (11.16 %)
Pathological Grading	I	94 (45.41 %)
	II	34 (16.43 %)
	III	79 (38.16 %)
	na	8 (3.72 %)
Margin status	negative	121 (61.11 %)
	positive	77 (38.89 %)
	na	17 (7.90 %)
Perineural invasion	negative	144 (73.10 %)
	positive	53 (26.90 %)
	na	18 (8.37 %)
Lymphovascular invasion	negative	154 (79.38 %)
	positive	40 (20.62 %)
	na	21 (9.76 %)
Extracapsular extension	negative	169 (88.48 %)
	positive	22 (11.52 %)
	na	24 (11.16 %)
Periparotid lymph node involvement	negative	167 (82.67 %)
	positive	35 (17.33 %)
	na	13 (6.04 %)

na = not available

**Table 2:** Histopathological grading for each histologic subtype of parotid cancer

Histology	Grade I (%)	Grade II (%)	Grade III (%)
Acinic Cell Carcinoma	53 (87)	2 (3)	6 (10)
Adenocarcinoma NOS	1 (5)	3 (16)	15 (79)
Adenoid Cystic Carcinoma	6 (35)	4 (24)	7 (41)
Basal Cell Adenocarcinoma	4 (80)	0	1 (20)
Carcinoma-ex Pleomorphic Adenoma	4 (22)	1 (6)	13 (72)
Mucoepidermoid carcinoma	26 (38)	24 (35)	18 (26)
Salivary duct Carcinoma	0	0	19 (100)

Positive surgical margins (i.e. tumor extending to the inked margin of specimen) were identified in 38.9 % (*n* = 77/198) of patients who underwent parotidectomy. Margin status was not available for 17 patients. Positive margins were reported in 12 patients with ACC, 10 patients with salivary duct carcinoma, and 6 patients with carcinoma ex-pleomorphic adenoma. Positive margins were noted in 34.8 % of patients (31/89) with grade 1 tumors, 32.3 % of patients (10/31) with grade II tumors, and 45.1 % of patients (32/71) with grade III tumors. Of the patients with positive margins, 36 (46.8 %) had their initial surgery performed at an outside center and were referred for further management. Eight of these patients underwent a repeat surgical resection as part of their management.

Perineural invasion (PNI) was reported in 53 (26.9 %) patients. Salivary duct carcinoma had the highest frequency (72.2 %, *n* = 13/18), followed by ACC (45 %, 9/20), adenocarcinoma (52.6 %, 10/19), carcinoma ex-pleomorphic adenoma (21.4 %, 3/14), MEC (16.4 %, 10/61), and acinic cell carcinoma (8.3 %, 5/58). The incidence of perineural invasion increased with histopathological grade. PNI was reported in 4.4 % (4/90) of grade I tumors, 26.7 % (8/30) of grade II tumors, and 55.7 % (39/70) of grade III tumors (*p*-value < 0.0001). Lymphovascular invasion (LVI) was reported in 40 (20.6 %) patients. It occurred most commonly in salivary duct carcinomas (61.1 %, *n* = 11/18), followed by adenocarcinoma (52.6 %, 10/19), carcinoma ex-pleomorphic adenoma (42.9 %, 6/14), acinic cell carcinoma (14.0 %, 8/57), ACC (10.5 %, 2/19), and MEC (5 %, 3/60). LVI was uncommon in grade I and grade II tumors (7.9 % and 6.7 %, respectively); however, it was frequently found in grade III tumors 31/40 (45.6 %) (*p*-value <0.0001).

Overall, 52 patients had nodal metastases. Thirty-five (67.3 %) of these patients had positive periparotid lymph nodes noted on final pathology, 29 (82.9 %) of which had extranodal extension. Periparotid nodal metastases were most commonly noted with salivary duct carcinomas (61 %; 11/18). This was followed in frequency by adenocarcinoma (22.7 %; 5/22), MEC (17.9 %; 12/67), acinic cell carcinoma (8.2 %; 5/61), carcinoma ex-pleomorphic adenoma (6.7 %; 1/15), and ACC (3.9 %; 1/26) (*p*-value < 0.0001). The incidence of periparotid nodal metastases increased with histological grade; 7.5 % of grade I tumors demonstrated periparotid nodal metastases, 11.8 % of grade II tumors, and 30 % of grade III tumors (*p*-value = 0.0003). Among the 35 patients with positive periparotid nodes 17 (56.7 %) were staged clinically as N0 (clinical nodal staging was not available for 5 patients). Moreover, of the 35 patients, with a positive periparotid node 38.8 % also had lateral neck nodal metastases, in contrast to 2.7 % of patients with negative periparotid nodes.

### Outcome

The mean and median follow-up durations for the entire cohort were 85.2 and 80.7 months, respectively. The mean and median follow-up durations of living patients were 101 and 102 months, respectively. At the time of last follow-up, 148 (68.8 %) patients were alive without disease, 7 (3.3 %) were alive with disease, and 4 (1.9 %) were lost to follow up (patients alive at last visit but with less than 2 months of follow-up). During the observation period, 36 patients (16.7 %) died of disease and 6 (2.8 %) died of other causes. The mean time to death was 39.9 months (range 1.77-129.64 months).

### Recurrence

During the study period 48 patients (22.3 %) developed a recurrence. Twelve developed local recurrence, 7 developed regional recurrence and 29 developed distant metastases. Eleven of these patients had two sites of recurrence, with the most frequent combination (*n* = 9, 4.2 %) being local and distant failure. The recurrence rate for node positive and node negative patients was 63.5 % and 23.0 %, respectively (*p* < 0.0001). The odds ratio of developing recurrent disease in the node positive compared to the node negative group was 5.80 (95 % CI 2.88-11.70). Furthermore, the incidence of distant metastases was significantly higher in the node positive group (30.8 %) than for the node negative group (6.35 %; *p*-value <0.0001). The odds ratio of detecting distant metastatic disease in the node positive group was 6.55 (95 % CI 2.59-18.56) compared to the node negative group. Patients with lymphovascular invasion had a significantly higher chance of having distant metastases (72.2 %) compared to those without lymphovascular invasion (39.1 %, *p* = 0.023).

### Survival

For the entire cohort, the mean and median follow-up time was 85 and 81 months (range 1–256 months), respectively. The 5- and 10-year OS was 80.6 % and 69.5 %, respectively. The 5- and 10-year DFS was 74.4 % and 62.4 %, respectively.

**Fig. 1:**
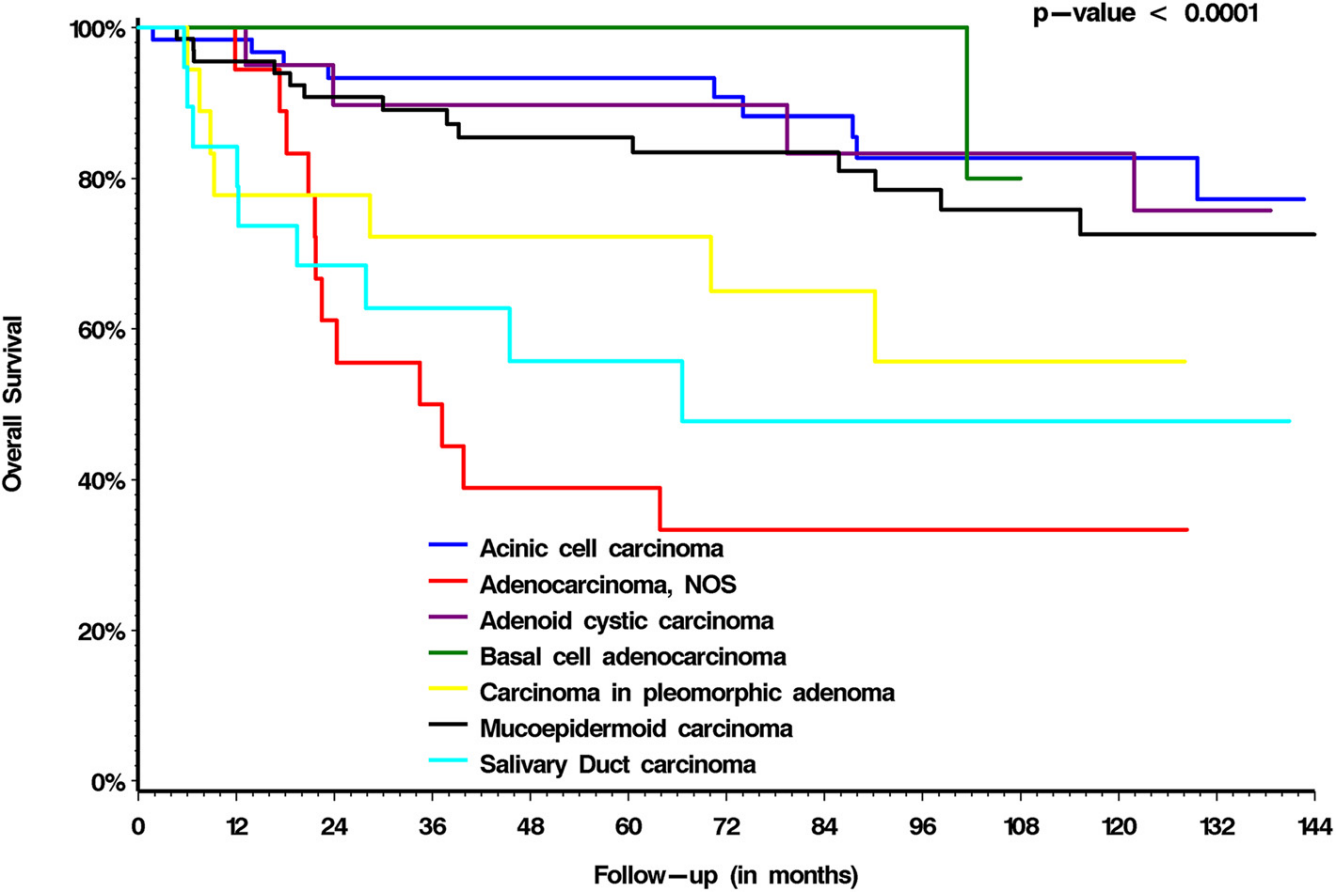
Kaplan-Meier curves for overall survival of 215 patients with parotid gland malignancies stratified by morphology

Histological subtype was a clear predictor of OS (Fig. 1) on univariable analysis. Other predictors of OS are summarized in Table 3. Significant predictors of OS on multivariable analysis are summarized in Table 4. Lymphovascular invasion, histopathologic grade, nodal status, age >60 and adjuvant radiation therapy was shown to be a significant predictor for overall survival. Patients who received radiotherapy postoperatively had a significant prolonged overall survival compared to patients who had no adjuvant radiotherapy (*p* = 0.007).

**Table 3:** Univariable analysis of overall-, and disease-free survival and clinicopathological parameters of patients with parotid gland carcinomas

	Univariable testing		
Variable	Overall survival	=	Disease-free survival
	*p*-value		
pT classification (T1/2 vs. T3/4)	<0.0001		<0.0001
pN status (N+ vs. N0)	<0.0001		<0.0001
Histopathological grade	<0.0001		<0.0001
Perineural invasion	<0.0001		<0.0001
Lymphovascular invasion	<0.0001		<0.0001
Extracapsular extension	<0.0001		<0.0001
Age <60y	<0.0001		0.0011
Histological subtype	<0.0001		0.0003
Periparotid node involvement	<0.0001		<0.0001
Positive Margins	0.37		0.08

pT and pN = pathological T and N classification

**Table 4:** Multivariable analysis of overall survival and clinicopathological parameters of patients with parotid gland carcinomas

Variable		Multivariable analysis for overall survival		
	*p*-value	Hazard ratio	95 % Confidence Interval	
Lymphovascular invasion	0.0020	3.217	1.532	6.755
Histopathological grade	0.0293	3.774	1.312	10.858
pN status	0.0430	1.060	1.002	1.122
Age >60	0.0352	1.990	1.049	3.775
Radiation yes/no	0.0056	0.272	0.106	0.696

pN = pathological N classification

**Fig. 2:**
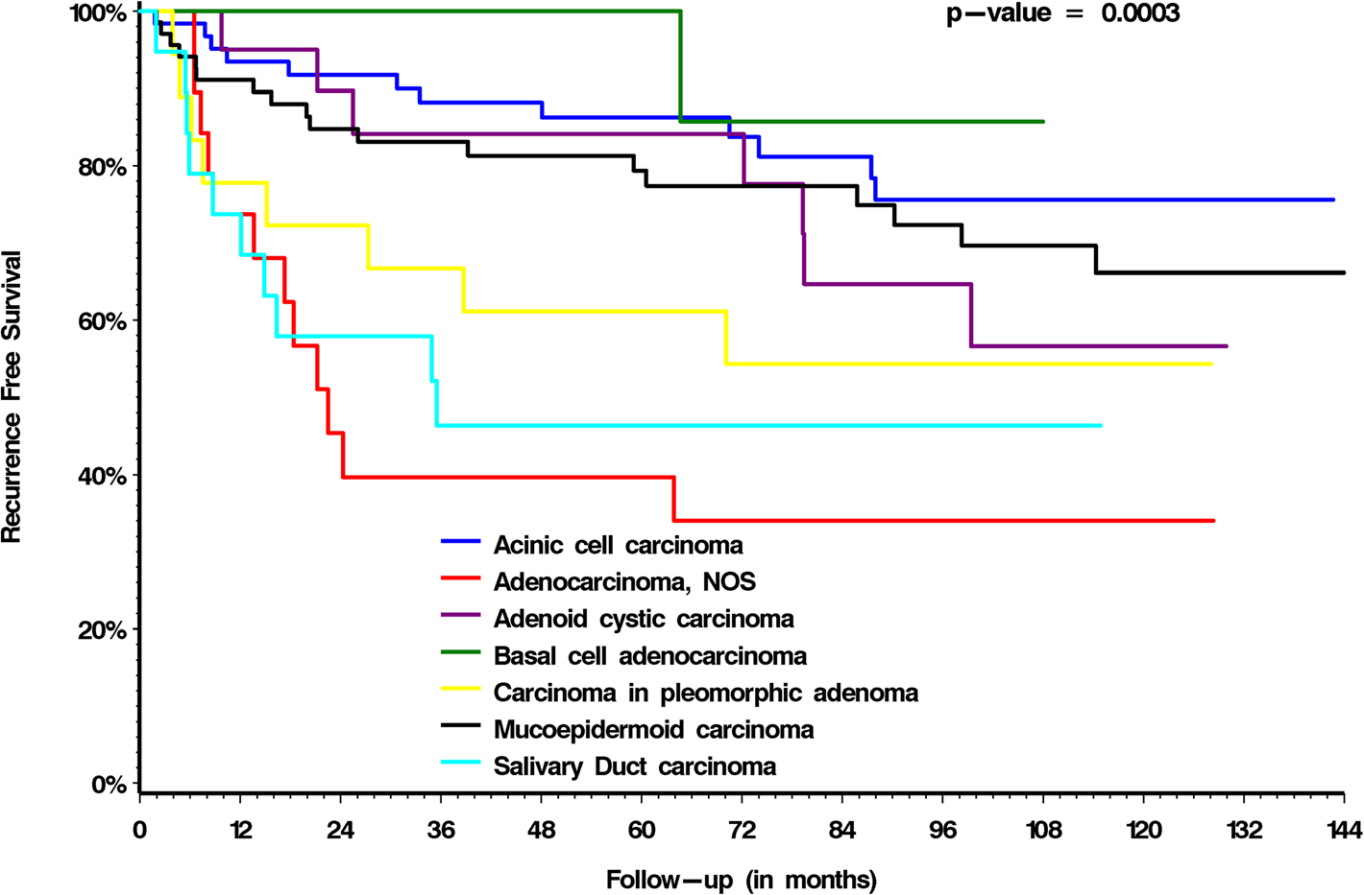
Kaplan-Meier curves for recurrence-free survival of 215 patients with parotid gland malignancies stratified by morphology

Predictors of disease-free survival (DFS) on univariable analysis are summarized in Table 3. Margin status was not a significant predictor of outcomes on UVA for either OS or DFS. Predictors of DFS on multivariable analysis were lymphovascular invasion, pathologic N classification, perineural invasion and age > 60 years (Table 5). Fig. 2 presents the Kaplan Meier DFS curves for the histologic subtype (*p* < 0.0001).

**Table 5:** Multivariable analysis of recurrence-free survival and clinicopathological parameters of patients with parotid carcinomas

Variable		Multivariable analysis for recurrence-free survival		
	*p*-value	Hazard ratio	95 % Confidence Interval	
Lymphovascular invasion	0.0482	1.953	1.005	3.794
pN status	0.0076	1.069	1.018	1.122
Perineural invasion	0.0040	2.526	1.345	4.745
Age >60	0.0447	1.749	1.013	3.018
Radiation yes/no	0.6233	0.822	0.375	1.798
Histopathological grade	NS			

pN = pathological N classification

NS = not significant

## Discussion

The heterogeneous nature of parotid gland malignancies, along with their diverse biological behavior and relative rarity can make their management very challenging. Knowledge of clinical and histopathologic prognostic factors is critical to making appropriate decisions regarding therapeutic options [4, 12, 13]. We sought to perform a review of outcomes of parotid cancer outcomes managed at a single tertiary care oncology center in the modern era and assess for predictors of outcome.

Our study cohort was comparable in demographic and clinicopathologic data to other recently published reports [7, 14]. Mucoepidermoid carcinoma and acinic cell carcinoma were the two most common variants. Patients with acinic cell, adenoid cystic and mucoepidermoid carcinoma had a better overall and recurrence-free survival compared to patients diagnosed with salivary duct carcinoma, carcinoma-ex-pleomorphic adenoma and adenocarcinoma. As has been demonstrated by the data in this study and others, patients can be stratified to high or low risk for survival according to their histology, as well as to their grade.

In concert with prior studies, we have also observed that patients with positive neck nodes, perineural, and lymphovascular invasion have lower overall and disease free-survival [7, 15, 16]. Similar to previous reports, patients younger than 60 years have a better disease-free survival than older patients. The explanation for this finding is unclear, although immunologic or other age-associated factors may play a role [17, 18].

Controversy exists as to the most appropriate management of the neck in patients with primary malignancies of the parotid. One of the complicating factors is the frequent lack of a preoperative histological subtype diagnosis. While most head and neck oncologists agree that elective neck dissection is warranted in those undergoing parotidectomy for high-risk or high-grade disease, stratification is often unknown at the time of primary surgery. Furthermore, in the setting of known malignancy, clinical nodal evaluation appears to significantly underestimate the true incidence of cervical nodal metastases [19]. Indeed, our study agreed with others that there is a significantly higher incidence of pathologic positivity in the neck then can be expected by exam or imaging. A recent meta-analysis has demonstrated that 23 % of patients with cN0 neck had positive disease [19]. However, there does appear to be an association between periparotid nodes and cervical lymphadenopathy. Klussman *et al.* [20] similarly noted of 36 patients with intraparotid nodes, 12 had positive cervical nodes (33 %). This raises the possibility that evidence of periparotid lymph node metastases may be a marker for more lateral neck nodal disease. In the current study lateral neck nodes were more common in those that had positive periparotid nodes than those that did not. In addition, almost half of the patients with a positive periparotid node were staged clinically as being N0. Thus, a decision for a lateral neck dissection may need to made at the time of surgery based on identifying a periparotid node and evaluating for metastases on frozen section analysis.

Multiple studies, including this one, have shown significantly worse outcomes with pathologically positive neck disease [3, 4, 7, 14]. In this cohort of patients, neck node positivity was a strong predictive factor for recurrent disease, in particularly for distant metastatic recurrence. In particular, 31 % of patients with nodal metastases have developed simultaneously distant metastases, primarily to the lungs. Looking at the nodal disease of all patients that have been included in this study we observed that subsequently the rate of patients who died of disease was significantly higher in the neck node positive group compared to patients with negative nodes in the neck. Furthermore, the disparity in outcomes may be underestimated due to a relatively high incidence of patients with occult cervical metastatic disease not undergoing elective neck dissection.

Lymphovascular invasion was also found to be a strong predictor of distant metastases and survival. On multivariable analysis with nodal metastases and grade included in the model LVI was still found to be a significant predictor of survival. One of the limitations was that the lymphatic and vascular invasion was not separated in pathology reports to determine whether one or both are associated with distant metastases and reduced survival [21, 22]. The high rate of distant metastases in patients with high-grade tumors, nodal metastases and LVI, points to the need for evaluating systemic therapies to be used alongside current treatment paradigms in patients with these high risk features.

As with most series facial nerve paralysis at presentation in our series was uncommon. While only 12 patients presented with facial paralysis 36 ultimately required facial nerve resection. Thus, patients with pre-operatively functioning nerves do need to be made aware of the possibility of nerve resection when there is evidence of nerve invasion or encasement intra-operatively.

Positive margins were reported in our series in almost 40 % of patients. This included patients that were managed elsewhere and referred in for further management, as well as cases where tumor was found to be extending to the margin of the specimen, in regions where the tumor is intimately related to the facial nerve. On analysis positive margin status was not a significant predictor of RFS. Almost all these patients would have received post-operative adjuvant therapy, thus highlighting the excellent control rates that can be achieved with adjuvant radiotherapy in the setting of microscopic positive margins. Lin and coworkers ^26^ compared the clinical outcome among 101 patients with salivary gland carcinomas after adjuvant radiotherapy. Although patients with positive margins had a shortened disease-free survival their clinical outcome was equal to those patients with negative margins [23].

While the inclusion of the diverse group of pathologies can be regarded as a limitation to the study we feel that the study provides an overview of the differences in the patterns of behaviour. In terms of outcomes, even within groups there is variability in biology based on tumor grade and thus tumor grade may provide more prognostic information than the type of parotid cancer.

## Conclusions

Our single institution experience has demonstrated that advanced age, lymph node status, perineural and lymphovascular invasion were the strongest predictors of oncologic outcome. Moreover we have shown that periparotid lymph node involvement has a major impact on disease-free and overall survival in patients with parotid carcinomas. The presence of periparotid lymph node involvement should alert the surgeon to the probability of advanced disease and the need for more aggressive treatment and follow up. Nevertheless, multi-institutional prospective studies are needed to further address this question. Currently, there is a phase II RTOG (1008) trial comparing adjuvant radiation versus chemoradiation in accruing patients with intermediate to high grade salivary gland carcinomas who have undergone curative intent surgical resection and are found to have the following risk factors for recurrence: T3-4, or N1-3 disease, or T1-2 N0 patients with positive or close (≤1 mm) microscopic margins of resection.
