# Erratum to: Outcome analysis of 215 patients with parotid gland tumors: a retrospective cohort analysis

**DOI:** 10.1186/s40463-015-0105-3

**Published:** 2015-12-15

**Authors:** Boban M. Erovic, Manish D. Shah, Guillem Bruch, Meredith Johnston, John Kim, Brian O’Sullivan, Bayardo Perez-Ordonez, Ilan Weinreb, Eshetu G. Atenafu, John R. de Almeida, Patrick J. Gullane, Dale Brown, Ralph W. Gilbert, Jonathan C. Irish, David P. Goldstein

**Affiliations:** Department of Otolaryngology-Head and Neck Surgery, Wharton Head and Neck Program, University Health Network, Princess Margaret Cancer Centre, Toronto, ON Canada; Department of Radiation Oncology, Princess Margaret Cancer Centre, University of Toronto, Toronto, ON Canada; Department of Pathology, University Health Network, Princess Margaret Cancer Centre, Toronto, ON Canada; Department of Biostatistics, University Health Network, Princess Margaret Cancer Centre, Toronto, ON Canada; Princess Margaret Hospital, Wharton Head and Neck Centre, 610 University Avenue, 3rd Floor, Toronto, M5G 2 M9 ON Canada

Unfortunately, the original version of this article [1] contained an error. The name of one of the authors was included incorrectly and it read “u Johnston” instead of “Meredith Johnston”.

The name has been updated in the original article and is also correctly included in full in this erratum.
